# Online Social Interaction, Neighborhood Perception, and the Mediating Role of Social Capital in Charitable Giving for Seriously Ill Patients: Cross-Sectional Study

**DOI:** 10.2196/85573

**Published:** 2026-06-26

**Authors:** Jie Chen, Junzhang Hu, Yang Yang, Fangjuan Du, Xuelian Li

**Affiliations:** 1 School of Geography and Environmental Sciences (School of Karst Science), Guizhou Normal University, Huaxi University Town, Guian New District, Guiyang, 550025, China, 86 13765109063; 2 School of Tourism and Air Service, Guizhou Minzu University, Guiyang, China; 3 School of International Tourism and Culture, Guizhou Normal University, Guiyang, China; 4 Faculty of Geography, Yunnan Normal University, Kunming, China

**Keywords:** online social interaction, bridging social capital, bonding social capital, donation, neighborhood perception, seriously ill patients

## Abstract

**Background:**

Online public welfare and donation-based crowdfunding have rapidly expanded, motivating individuals to engage in charitable giving through digital platforms. However, the influence of online social interactions and offline neighborhood perceptions on donation behavior remains underexplored.

**Objective:**

This study investigates how online social interactions influence individuals’ intentions to donate to patients with serious illness, focusing on the mediating roles of bridging and bonding social capital and the moderating effect of neighborhood perception. It integrates online and offline social contexts to explain donation behavior.

**Methods:**

Data were collected from individuals recruited through the “3C” (Caring for Children with Cancer) Health Care Volunteer Community in Guizhou, China. Validated scales were used to measure online social interaction, social capital, donation intention, and neighborhood perception. A total of 450 valid responses were obtained between August and October 2023, with a 91.46% response rate.

**Results:**

Online social interaction was positively associated with donation intention (*β*=0.37, *P*<.01). It significantly predicted both bridging social capital (*β*=0.45, *P*<.01) and bonding social capital (*β*=0.54, *P*<.01), which in turn were positively associated with donation intention (*β*=0.48 and *β*=0.54, respectively, *P*<.01). Bridging social capital partially mediated this relationship, whereas bonding social capital showed a stronger mediating effect. Neighborhood perception significantly moderated the relationship between online social interaction and both bridging (*β*=0.17, *P*<.01) and bonding social capital (*β*=0.18, *P*<.01). Furthermore, moderated mediation analysis showed that the indirect effects were stronger at higher levels of neighborhood perception (bridging: *β*=0.278; bonding: *β*=0.340) than at lower levels (bridging: *β*=0.150; bonding: *β*=0.181). The indices of moderated mediation were significant for both bridging (*β*=0.080, 95% CI 0.021-0.142) and bonding social capital (*β*=0.100, 95% CI 0.040-0.162).

**Conclusions:**

Online social interaction was associated with increased donation intentions to patients with serious illness via bridging and bonding social capital, with bonding ties fully and bridging ties partially mediating the effect. Neighborhood perception strengthened these pathways, indicating that offline social context can amplify the impact of online engagement on charitable giving. These findings highlight the importance of integrating digital interaction and offline social context in promoting health-related charitable giving.

## Introduction

### Background

The rapid expansion of digital technologies has transformed health-related charitable giving into an increasingly prominent component of the broader digital health (eHealth) ecosystem [1,2]. Online medical crowdfunding platforms, as a form of digitally mediated health support, enable individuals to mobilize financial resources for patients in need [3]. For example, Tencent’s public welfare platform claimed that user donations reached CNY 5.6 billion (approximately US $790 million) in 2022, involving more than 127 million donors (Tencent Public Welfare Platform, 2023). This indicates the expanding scale of digital charity activity. However, such expansion does not necessarily result in equal assistance outcomes. Evidence suggests that access to and effectiveness of digital health–enabled charitable support remain uneven [4]. Patients with serious illnesses, particularly in less-developed regions such as Western China, often receive comparatively less financial support through crowdfunding platforms [5-7]. Recent empirical evidence indicates that regional socioeconomic disparities and the “digital divide” significantly constrain fundraising performance, as individuals in these areas often lack the extensive online social networks required to mobilize sufficient donations [8]. This highlights a critical challenge within digital health systems: although technological platforms expand opportunities for participation, they do not automatically translate into equitable health-related support outcomes. Understanding what drives individuals to engage in donation behavior within these digitally mediated environments is therefore essential. Prior research suggests that charitable donations can enhance civic awareness and social cohesion while enabling individuals to express prosocial values [9]. However, recent national reports indicate a decline in overall social donations. For instance, the 2023 China Philanthropy Development Report revealed a 4.63% decrease compared to previous years, suggesting increasing uncertainty in digital charitable participation. To better explain these disparities, it is necessary to move beyond structural explanations and examine the relational mechanisms embedded in digital platforms. In particular, the role of online social interaction as a key driver of donation behavior remains insufficiently understood.

Though the mechanisms underlying public donation have been widely studied, the influence of individual cyberspace engagement on donation intention remains insufficiently understood. Previous research has distinguished between endogenous and exogenous determinants of donation behavior, including enjoyment and peer influence [10]. However, empirical evidence on how online social interactions shape willingness to donate to patients with serious illness remains limited [11]. Existing research has primarily focused on charitable giving and broader forms of prosocial engagement in general social networking contexts [12,13], with less attention paid to health care–related charitable contexts. In such situations, donation decisions are often influenced by trust, empathy, and emotional vulnerability [14,15]. In addition, the role of offline neighborhood perception in shaping digital prosocial participation has received little systematic attention [16]. The above indicates that the mechanisms underlying the relationships between online social interactions, social capital, donation intentions, and perceptions of neighborhood in health care settings warrant further investigation. To address this, this study focuses on active forms of online social interaction, including direct communication and engagement behaviors such as liking and commenting [17]. These interactions reflect emotional support and identification with charitable activities, thereby facilitating patient care and communication within online communities [18,19].

From a social capital perspective, charitable donation can be understood as a form of resource exchange embedded in social relationships [20,21]. Accordingly, this study adopts a relational perspective to examine how online social interaction influences donation intention through bridging and bonding social capital, and how this process is conditioned by neighborhood perception as an offline contextual factor [22,23]. Focusing on health care volunteer communities, this study highlights the distinct roles of bridging and bonding social capital in serious illness donation contexts. Bridging ties facilitate information diffusion and network expansion, whereas bonding ties are particularly salient in health care–related settings due to their role in fostering trust, empathy, and emotional support [14,24]. This study integrates online social interactions, social capital, and perceptions of offline neighborhood, thereby expanding the application of social capital theory in the field of digital health philanthropy and deepening our understanding of the mechanisms underlying digital altruistic behavior.

### Literature Review and Hypotheses: Social Capital Theory

Social capital has been conceptualized in various ways, but is generally understood as resources embedded in social networks and accessible through social relationships [24]. Despite differing definitions, the core concept of social capital involves social networks or structures and the resources they provide. Social capital can be manifested through individuals’ social networks and is commonly divided into 2 dimensions: bridging and bonding social capital [20]. Bridging social capital emphasizes superficial and diverse relationships, typically involving weak ties between people from different backgrounds. It broadens social horizons and facilitates information exchange. Bonding social capital involves strong ties between family or close friends and reflects emotional or practical support from individuals with similar backgrounds or deep connections. Social networks comprise channels of information and emotional bonds, embedding the norms and value system that promote good behavior among individuals, forming social capital [24].

Interactions in cyberspace contribute to the accumulation of online social capital [23], which is obtained by connecting with others through information communication technologies [22]. A key feature of the internet is that it allows people to make new connections online or maintain offline relationships [24]. As such, it can be viewed as a tool for maintaining relationships or achieving goals [25]. From a social capital perspective, internet use serves as an important mechanism for accumulating social resources and promoting prosocial behaviors such as community participation and helping others [11]. It can also influence willingness to reciprocate [26], sense of community [23], and continuance intention [27] to promote and stimulate personal behaviors. More broadly, social capital fosters trust, cooperation, and reciprocity, which in turn facilitate collective action and shape individual decision-making processes [28-30]. These mechanisms suggest that social capital functions as a relational resource shaping behavioral intentions in online and offline contexts.

### Online Social Interaction and Intention to Donate

Social interaction is a key factor in facilitating information exchange [31] and provides a foundational condition for promoting prosocial behavior (eg, charity or donation). Social media use is associated with civic engagement, life satisfaction, and social trust [32,33]. It also provides informational and emotional support, and resources for self-regulation [10,34]. In terms of use patterns, researchers distinguish between passive and active social media use. Passive use refers to content consumption, while active use emphasizes interactive engagement and mutual communication [17]. Active social interaction enables users to create a positive experience through encouragement, affirmation, and mutual support [35].

Given the virtual nature of the internet, online interactions weaken an individual’s personal identity and enhance group identity. It gives people a sense of belonging when connecting or sharing common experiences [23], thus encouraging them to continue commenting on each other’s content or sharing experiences [18]. Positive interactions also create social pressure for individuals to engage in collective activities [35]. From a collective standpoint, many positive behaviors arise from the pressure of social norms in social interactions [36]. Personal interactions highlight and reinforce social norms, thereby guiding individual behavior [37]. Studies have further shown that it is positively associated with charitable giving and donation behavior [38,39]. Therefore, we formulated the following hypothesis: online social interaction is positively associated with intention to donate (H1).

### Mediation of Bridging Social Capital

Social media encourages the exchange of information, advice, and ideas based on the needs and resources of common interest [40], allowing users to obtain the relevant social support [25]. Through low-cost and convenient communication, online interaction strengthens weak ties among individuals and fosters the development of bridging social capital [23,24]. For example, Ellison et al [34] found that social media use can facilitate and predict bridging social capital. Hence, we hypothesized: online social interaction positively affects bridging social capital (H2a).

The benefits of bridging social capital can be reflected in its connection with external assets and diffusion of information [20]. After the formation of networks with weak ties, information flows faster, and connections become closer [12]. Bridging social capital influences users’ attitudes and perceived credibility in stimulating individual engagement [28]. A bridging social capital perspective enables an increase in community engagement and collective cooperation based on large and diverse networks [41]. Visible donations incentivize donors to improve their status or maintain relationships [38], and individuals are accustomed to comparing their donations with those of others [42]. Therefore, we posited: bridging social capital positively affects intention to donate (H2b).

The internet creates an environment in which individuals can communicate with others, which benefits the expansion and extension of social networks [43]. Network ties reduce the time and investment required to gather information and knowledge, which in turn generates norms, enables individuals to participate in and contribute to activities, and increases their willingness to maintain relationships [44]. Research has clarified that bridging social capital accumulated through online interactions also leads to positive psychological outcomes that motivate individuals to donate [32,43]. Therefore, scope-based bridging social capital provides a large stock of social resources to guide people to make charitable donations [21]. Combining H1, H2a, and H2b, we proposed: bridging social capital mediates the positive relationship between online social interaction and intention to donate (H2).

### Mediation of Bonding Social Capital

Bonding social capital is rooted in emotional and substantive support derived from close and intimate relationships formed through strong social ties [24]. Internet use can supplement offline relationships with family or close friends [45]. Interactions within a virtual platform strengthen the bonds between members [23], and online social connections can evolve into lasting and strong relationships over time [46]. Studies have suggested a direct relationship between online interactions and bonding social capital. For example, social networking platforms provide new opportunities for maintaining and strengthening strong ties [47], expanding family and friend networks while offering emotional support [48]. In addition, individuals tend to interact with similar others online, which facilitates the development of trust-based and emotionally secure relationships [49]. Thus, we hypothesized: online social interaction is positively associated with bonding social capital (H3a).

Bonding social capital is characterized by strong ties and has a considerable impact on outcomes and human behavior, such as emotional support, collective action, and mobilizing solidarity [24]. Personal involvement in charitable organizations plays a significant role in generosity and donations [50]. Additionally, strong family and neighborhood social networks positively affect donations [51]. We can, thus, infer that volunteers who have close contact with patients or experience of caring for them may have strong emotions and expectations, build bonding social capital, and promote charitable giving. Therefore, we posited: bonding social capital positively affects the intention to donate (H3b).

Compared to bridging social capital, bonding social capital may play a more important role in health care–related donation contexts, as serious illness donations often involve trust, empathy, and emotional vulnerability [14,52]. In such situations, individuals are more likely to rely on relationships based on emotional support and trust rather than on relationships based on weak ties [24]. Therefore, bonding social capital may have a stronger influence on donation intention than bridging social capital.

Social bonding mediates the relationship between online social interactions and an individual’s intentions [27]. Online participation can create the perception that others support community goals. This may lead users to form common norms and pursue the same goals [33]. Membership and friendships in the community correlate with the frequency of donations [53]. Trust, competence, discipline, and kindness are built on substantial interactions among communities and form bonding social capital, thus promoting good behavior. Combining H1, H3a, and H3b, we proposed: bonding social capital mediates the positive relationship between online social interaction and intention to donate (H3).

### Moderating Role of Neighborhood Perception

Social cohesion and donation networks suggest that prosocial behaviors are embedded within broader social environments and relational contexts [16]. Neighborhoods, as localized social settings, reflect varying levels of cohesion, safety, and interpersonal connectedness [54]. In this study, neighborhood perception is conceptualized as individuals’ subjective evaluation of social cohesion, safety, and relational quality within their local environment. Prior research suggests that individuals in more advantaged neighborhoods tend to display higher levels of social cohesion than those in disadvantaged neighborhoods, leading to differences in social attitudes and behavioral outcomes [55]. Such variation reflects how individuals interpret and respond to their surrounding social environments. Rather than representing a single mechanism, neighborhood perception operates as a contextual condition that shapes social cognitive processes. Specifically, it may influence the formation of online social capital through a norm-based mechanism, in which individuals become more sensitive to social expectations and reputational concerns when perceiving their neighborhood environment as socially salient, and a contact-based mechanism, in which higher neighborhood perception facilitates trust and interpersonal interaction [56,57].

In the context of norm-based mechanisms, symbolic threats refer to potential challenges to morals, beliefs, and norms caused by groups with different value systems. This can involve the risk of loss of face or honor and potentially hurt an individual’s self-identity or self-esteem [58]. According to the social capital theory, bridging social capital can trigger individuals to generate cognitive responses or stress behaviors and make themselves conform to related norms [59]. Accordingly, individuals with stronger neighborhood perception may experience greater normative awareness during online social interactions, which facilitates the development of bridging social capital. Individuals with higher neighborhood perception are more sensitive to local social norms and reputational cues, which may facilitate broader social interactions and the development of bridging social capital [60]. We propose that individuals with higher neighborhood perception are more likely to develop stronger bridging social capital through social cohesion and interaction. Thus, we proposed: neighborhood perception moderates the direct effect of online social interaction on bridging social capital (H4).

From a contact-based perspective, neighborhood perception plays an important role in promoting social integration and fostering harmonious social relationships [61]. The contact mechanism states that increased opportunities for interpersonal interaction within neighborhoods enhance mutual understanding, reduce bias, and promote social cohesion [54]. Social networks in more cohesive neighborhoods are more likely to be channels for information circulation and emotional bonding. Interpersonal contact reduces bias, improves intergroup relationships, and increases empathy and understanding [62]. As individuals develop stronger perceptions of neighborhood cohesion, they are more likely to internalize a sense of belonging, trust, and a sense of community [63]. These relational and affective processes foster deeper interpersonal connections and reinforce strong-tie networks. Therefore, stronger neighborhood perception is expected to enhance bonding social capital through the contact-based mechanism, particularly in the context of online social interaction. Thus, we proposed: neighborhood perception moderates the direct effect of online social interaction on bonding social capital (H5).

**Figure 1.**
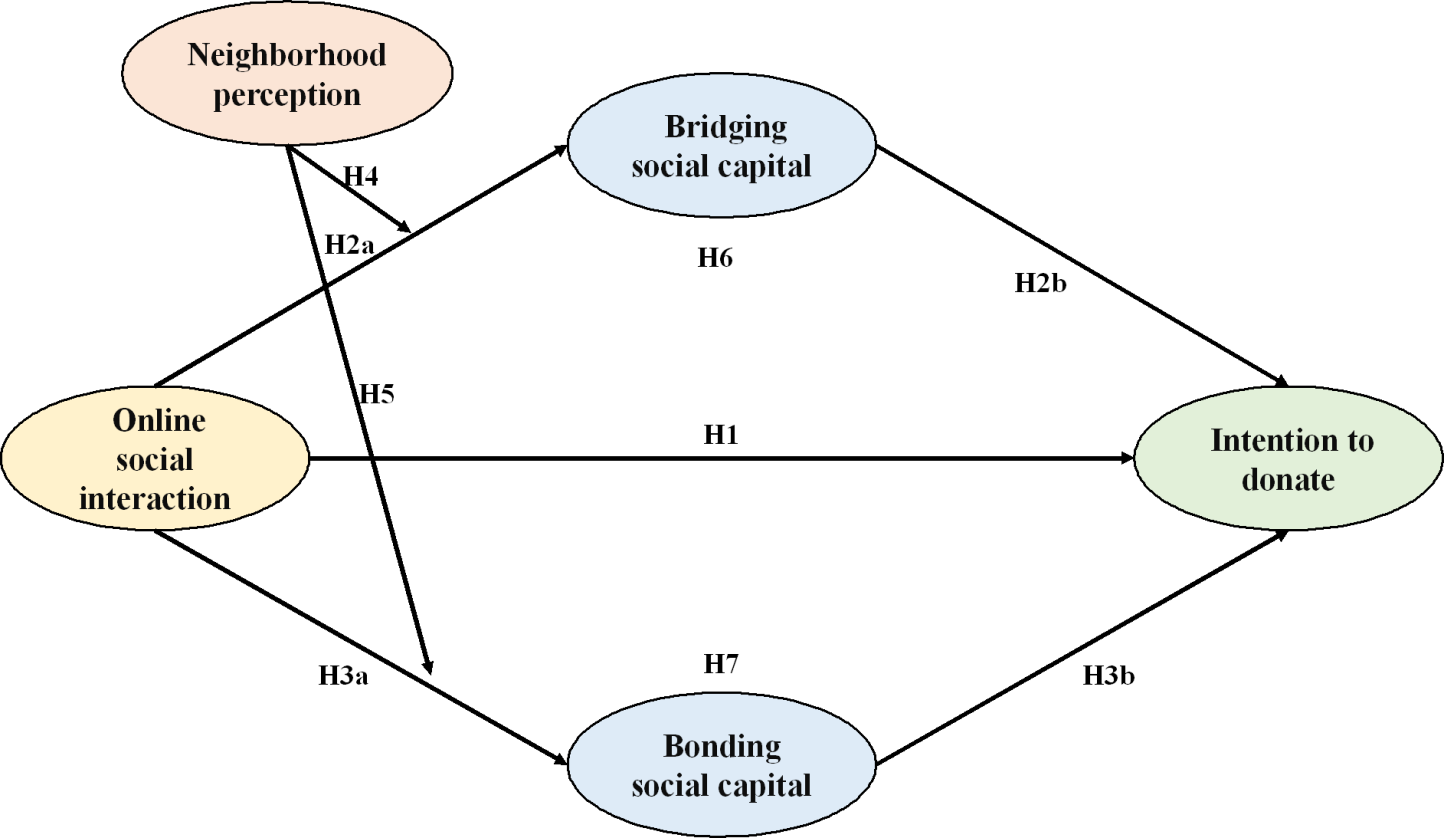
Conceptual model of the study.

Neighborhoods capture the social environment and, consequently, affect individuals’ prosocial behavior in the real world [64]. Individuals embedded in neighborhoods with stronger neighborhood ties produce stronger sources of social influence [16]. Building on the dual-mechanism framework, neighborhood perception is expected to strengthen the indirect effects of online social interaction on donation intention through both bridging and bonding social capital. From a norm-based perspective, higher neighborhood perception heightens sensitivity to social norms and reputational expectations, promoting engagement in broader weak-tie networks and thereby reinforcing the indirect effect via bridging social capital [16]. From a contact-based perspective, stronger neighborhood perception enhances trust, interaction, and a sense of belonging, fostering emotionally supportive ties and strengthening the indirect effect via bonding social capital [61]. Taken together, these complementary mechanisms suggest that higher neighborhood perception amplifies both indirect pathways linking online social interaction to donation intention. Based on the above mediation hypotheses, we proposed: neighborhood perception moderates the indirect effect of online social interaction on the intention to donate via bridging social capital, such that the indirect effects are stronger when neighborhood perception is higher (H6) and neighborhood perception moderates the indirect effect of online social interaction on the intention to donate via bonding social capital, such that the indirect effects are stronger when neighborhood perception is higher (H7). Figure 1 presents the conceptual model of this study. The model illustrates the direct effect of online social interaction on intention to donate (H1), 2 parallel mediation pathways through bridging and bonding social capital (H2a-H2b; H3a-H3b), the moderating effects of neighborhood perception on the relationships between online social interaction and both types of social capital (H4, H5), and the moderated mediation effects (H6, H7). In the figure, oval shapes represent latent constructs, arrows indicate hypothesized relationships, and H denotes a hypothesis.

## Methods

### Study Setting

This study was conducted within the context of the “3C” (Caring for Children with Cancer) Health Care Volunteer Community, a health care support initiative founded by university faculty members and involving serious illness care and volunteer community engagement. The 3C community operates primarily in Guizhou, China, through collaborations with hospitals and rehabilitation organizations. The 3C community has developed both online and offline engagement platforms, including WeChat Moments and Public Channels (Tencent), to facilitate communication and dissemination of charitable activities. Regular volunteer activities are conducted on weekdays in the Children’s Hematology Department of Guiyang Maternal and Child Health Hospital, including games, painting, handicrafts, education, and music for children with leukemia. The 3C collaborates with public service centers to conduct follow-up care for patients with serious illnesses and organize nature-based therapeutic activities for children with cancer and their families. In addition, the community operates an online crowdfunding initiative (“Qiantong Health”) to support charitable fundraising for children with serious illnesses. Through ongoing online and offline care activities, the community gradually attracted broader participation beyond core volunteers, including peripheral supporters and individuals exposed to its charitable initiatives or online content. Therefore, these online volunteer care communities, together with their extended social networks, represent typical cases that reflect our research aims.

### Participants and Recruitment

Participants were recruited from the “3C” Health Care Volunteer Community and its extended social networks. A purposive sampling strategy was used to ensure that participants were engaged in or influenced by volunteer care activities related to serious illness. Eligible participants included individuals with varying levels of connection to the 3C community, including (1) those directly involved in volunteer-related activities, such as hospital-based volunteer services, online community interactions, or follow-up care activities; and (2) broader community members who were exposed to the 3C community through its online content, charitable initiatives, or related support activities. Potential participants were approached through multiple channels, including WeChat groups, online community platforms, and face-to-face invitations during ongoing volunteer activities at the hospital. Individuals who expressed interest were screened for eligibility based on their level of engagement with or connection to the volunteer care network. All eligible participants were informed about the purpose and procedures of the study. Participation was voluntary, and informed consent was obtained prior to data collection.

### Data Collection

Data collection was divided into 2 phases: the precollection stage (August 25‐30, 2023) and the formal data collection stage (September 23-October 8, 2023). In phase 1, 200 questionnaires were distributed and 176 were collected, with an 88% response rate. We eliminated items with low reliability and modified any ambiguous items. A formal questionnaire was distributed online and offline in phase 2. To ensure the relevance of the survey context, respondents were required to have some degree of familiarity with or exposure to health care volunteer activities or related charitable initiatives associated with the 3C community. These respondents had been identified prior to the commencement of the study. A total of 492 questionnaires were distributed; 488 were collected, and 450 were valid, for a response rate of 91.46%. The statistical information of the samples is shown in Table 1. To reduce potential bias, anonymity was ensured and items were presented in a mixed order.

**Table 1.** Demographic characteristics of participants (N=450).

Category and item	Value, n (%)
Sex	
Male	166 (36.9)
Female	284 (63.1)
Age (years)	
≤20	121 (26.9)
21‐30	266 (59.1)
31‐40	34 (7.6)
41‐50	19 (4.2)
51‐60	7 (1.6)
>61	3 (0.7)
Education	
High school or below	27 (6.0)
Junior college	61 (13.6)
Bachelor's degree	252 (56.0)
Graduate degree	110 (24.4)
Residence	
Urban	225 (50.0)
Rural	225 (50.0)
Political affiliation	
CPC^a^ member	78 (17.3)
Non-CPC member	372 (82.7)
Marital status	
Unmarried	378 (84.0)
Married	63 (14.0)
Divorced	9 (2.0)
Monthly income, CNY (US $ equivalent)	
≤3000 CNY (≤US $425)	269 (59.8)
3000‐6000 CNY (US $425-$851)	93 (20.7)
6000‐8000 CNY (US $851-$1135)	45 (10.0)
8000‐10,000 CNY (US $1135-$1418)	22 (4.9)
≥10,000 CNY (≥US $1418)	21 (4.7)

a CPC: Communist Party of China.

### Measurements

In this study, we used measures drawn from previous research and previously validated instruments. A standard 5-point Likert-type response format was used, ranging from strongly disagree (1) to strongly agree (5). To ensure linguistic and conceptual equivalence in the Chinese context, a translation and back-translation procedure was conducted. First, the original English items were translated into Chinese by a bilingual researcher. Then, an independent bilingual expert back-translated the Chinese version into English. Discrepancies between the original and back-translated versions were discussed and resolved to ensure semantic consistency. A pilot test was also conducted to assess the clarity and cultural appropriateness of the translated items, and minor revisions were made accordingly.

Online social interaction was measured using 5 items adapted from established online interaction scales that have demonstrated good reliability in previous digital communication research [17,23]. We adopted a 10-item scale adapted from previously validated measures to assess online social capital, including bridging social capital (5 items) and bonding social capital (5 items) [23,24]. A 3-item scale measuring behavioral intentions to donate was adapted from Thomas and Jadeja [65], which has been used to assess charitable giving intentions. Neighborhood perceptions were measured using 7 items adapted from a well-validated scale assessing residents’ perceptions of neighborhood trust, safety, and physical conditions [66]. Finally, demographic variables, including sex, age, income, and census register, were included as control variables based on prior research [67].

### Ethical Considerations

This study was conducted in accordance with the Declaration of Helsinki and was approved by the Ethics Committee of Guizhou Normal University (approval number 202407001). All participants were informed about the purpose and procedures of the study, and participation was voluntary. Informed consent was obtained from all participants prior to data collection. Given the minimal-risk nature of the study, verbal consent was considered appropriate. All data were collected anonymously and used solely for research purposes.

## Results

### Data Normality and Common Method Bias Analysis

Data normality was assessed using skewness (−0.876 to 1.311) and kurtosis (−0.810 to 1.130). According to Kline [68], these values indicate acceptable normality. To examine the potential impact of common method bias (CMB), we first conducted Harman’s single-factor test following Anderson and Gerbing [69]. The results revealed that 5 factors with eigenvalues greater than one were extracted, and the first factor accounted for 38.55% of the total variance, suggesting that CMB was unlikely to be a serious concern [70]. Given that Harman’s single-factor test has been widely criticized as an insufficient diagnostic technique, we further applied a latent method factor approach following Podsakoff et al [70]. Specifically, a common latent factor (CLF) was incorporated into the measurement model, with all observed indicators loading onto both their theoretical constructs and the latent method factor, while constraining the method factor to be uncorrelated with the substantive constructs. Minor item refinements were applied to ensure model stability. The results indicated that the model fit remained acceptable after including the CLF (*χ*²_257_=785.675, Comparative Fit Index=0.938, Tucker-Lewis Index=0.927, Root Mean Square Error of Approximation=0.068, Standardized Root Mean Square Residual=0.089). Moreover, the inclusion of the CLF did not substantially alter the magnitude or significance of the factor loadings, suggesting that CMB is unlikely to pose a serious threat to the validity of the findings.

### Confirmatory Factor Analysis

Before testing the hypotheses, we conducted a series of confirmatory factor analyses with maximum-likelihood estimation in Mplus 7.0 (Muthén & Muthén) to examine the distinctiveness of our study’s constructs [71]. Table 2 shows that the 5-factor measurement model demonstrated an acceptable fit for the data (*χ*²_258_=803.47, Comparative Fit Index=0.94, Tucker-Lewis Index=0.93, Root Mean Square Error of Approximation=0.07, and Standardized Root Mean Square Residual=0.08). All factor loadings were significant. The 5-factor model demonstrated a significantly better fit than alternative models, thereby supporting the discriminant validity of the constructs [72].

**Table 2.** Confirmatory factor analysis results (N=450).

Model	Chi-square (*df*)	*P* value	CFI^a^	TLI^b^	RMSEA^c^	SRMR^d^
5-factor model: OSI^e^, BSC^f^, OSC^g^, ITD^h^, EP^i^	803.47 (258)	<.001	0.94	0.93	0.07	0.08
4-factor model 1: OSI, BSC+OSC, ITD, EP	1808.82 (269)	<.001	0.82	0.80	0.11	0.11
4-factor model 2: OSI, BSC, OSC, ITD+EP	2509.84 (269)	<.001	0.74	0.71	0.14	0.12
4-factor model 3: OSI+EP, BSC, OSC, ITD	2794.32 (269)	<.001	0.70	0.67	0.14	0.14
3-factor model 1: OSI+EP+ITD, BSC, OSC	3512.76 (272)	<.001	0.62	0.58	0.16	0.14
3-factor model 2: OSI+BSC+OSC, EP, ITD	2561.12 (272)	<.001	0.73	0.70	0.14	0.12
2-factor model: OSI+BSC+OSC, ITD+EP	3594.23 (274)	<.001	0.61	0.57	0.16	0.13
1-factor model: OSI+BSC+OSC+ITD+EP	4597.33 (275)	<.001	0.49	0.44	0.19	0.14

a CFI: Comparative Fit Index.

b TLI: Tucker-Lewis Index.

c RMSEA: Root Mean Square Error of Approximation.

d SRMR: Standardized Root Mean Square Residual.

e OSI: online social interaction.

f BSC: bridging social capital.

g OSC: bonding social capital.

h ITD: intention to donate.

i EP: neighborhood perception.

### Descriptive Statistics

All items are reported in Table 3. The factor loadings of these constructs were significant (0.53 to 0.97 level) [73]. The constructs had adequate and satisfactory convergent validity, as all the average variance extracted (AVE) values were higher than the threshold of 0.50 [74]. The Cronbach α values of these constructs and composite reliability were higher than 0.86. This illustrates strong internal consistency based on all measures. The means, SDs, reliabilities, zero-order Pearson correlations, and square roots of the AVEs are presented in Table 4. Online social interaction was positively correlated with bridging social capital (*r*=0.46, *P*<.01), bonding social capital (*r*=0.55, *P*<.01), and intention to donate (*r*=0.37, *P*<.01). Both bridging social capital (*r*=0.54, *P*<.01) and bonding social capital (*r*=0.55, *P*<.01) were positively correlated with intention to donate. Moreover, bridging social capital and bonding social capital were positively correlated (*r*=0.69, *P*<0.01), providing preliminary support for our hypotheses. In addition, the square roots of the AVEs for all constructs exceeded their interconstruct correlations, further supporting discriminant validity.

**Table 3.** Reliability and validity of the constructs.

Constructs and items	Standardized factor loading	Cronbach α	CR^a^	AVE^b^
Online social interaction		0.86	0.86	0.56
I like comments frequently on the Internet.	0.63			
I reply to comments frequently on the Internet.	0.91			
I participate in discussions frequently on the Internet.	0.93			
I often chat privately with strangers on the Internet.	0.59			
I often leave a message on others’ profiles on the Internet.	0.61			
Bridging social capital		0.92	0.92	0.71
Interacting with people in online charity communities makes me interested in things that happen outside of my school or work.	0.78			
Interacting with people in online charity communities makes me want to try new things.	0.90			
Interacting with people in online charity communities makes me curious about other places in the world.	0.88			
Interacting with people in online charity communities makes me interested in what people unlike me are thinking.	0.85			
Interacting with people in online charity communities reminds me that everyone in the world is connected.	0.79			
Bonding social capital		0.87	0.87	0.58
There are several people in online charity communities I trust to help solve my problems.	0.78			
There is someone in the online charity community that I feel comfortable talking to about intimate personal problems.	0.76			
There is someone in online charity communities I can turn to for advice about making very important decisions.	0.84			
When I feel lonely, there are several people in online charity communities I can talk to.	0.77			
If I needed an emergency loan of 4000 RMB, I know someone in the online charity community I can turn to.	0.64			
Neighborhood perception		0.87	0.87	0.51
I think my neighborhood is safe during the day.	0.58			
I think my neighborhood is safe at night.	0.60			
I could call my neighbors for help.	0.53			
My neighbors and I trust each other.	0.63			
The buildings and roads in my neighborhood are well-maintained.	0.84			
I feel good about my home or neighborhood.	0.88			
My neighborhood is kept clean.	0.84			
Intention to donate		0.95	0.95	0.87
When I see crowdfunding online platforms for seriously ill patients, I would like to donate money to them through online charitable organizations.	0.90			
When I see crowdfunding online platforms for seriously ill patients, I intend to donate money to them through online charitable organizations.	0.97			
When I see crowdfunding online platforms for seriously ill patients, I will donate money to them through online charitable organizations.	0.92			

a CR: composite reliability.

b AVE: average variance extracted.

**Table 4.** Correlations, means, and SDs of all the study variables (N=450).

	Sex	Age	Income	Education	Census register	Political identity	Marital status	OSI^a^	BSC^b^	OSC^c^	ITD^d^	EP^e^
Sex	—^f^											
Age	−0.02	—										
Income	−0.09	0.39^g^	—									
Education	−0.05	−0.06	−0.08	—								
Census register	−0.01	−0.23^g^	−0.07	−0.01	—							
Political identity	0.02	−0.20^g^	−0.13^g^	−0.22^g^	−0.01	—						
Marital status	0.05	0.59^g^	0.33^g^	−0.26^g^	−0.16^g^	−0.05	—					
OSI^a^	−0.01	−0.05	−0.02	−0.14^g^	−0.05	0.01	0.02	0.75^i^				
BSC^b^	0.05	−0.04	−0.04	−0.08	−0.06	−0.04	−0.04	0.46^g^	0.84			
OSC^c^	−0.06	−0.04	−0.08	−0.14^g^	−0.02	0.02	−0.02	0.55^g^	0.69^g^	0.76		
ITD^d^	0.09	−0.01	−0.03	−0.12^h^	0.01	−0.01	−0.01	0.37^g^	0.54^g^	0.55^g^	0.93	
EP^e^	−0.11^h^	0.07	0.08	−0.05	−0.05	−0.02	0.01	0.27^g^	0.26^g^	0.40^g^	0.44^g^	0.71
Mean (SD)	1.63 (0.48)	1.96 (0.87)	1.74 (1.12)	2.99 (0.79)	1.50 (0.50)	1.83 (0.38)	1.18 (0.43)	2.58 (0.84)	2.99 (0.99)	2.58 (0.95)	3.34 (1.01)	3.50 (0.80)

a OSI: online social interaction.

b BSC: bridging social capital.

c OSC: bonding social capital.

d ITD: intention to donate.

e EP: neighborhood perception.

f Not applicable.

g Statistically significant at the .01 level

h Statistically significant at the .05 level

i Italicized diagonal values represent the square roots of average variance extracted.

### Hypotheses Testing

We conducted a regression analysis to test H1. The results indicated that online social interaction was positively correlated with intention to donate (*β*=0.37, SE 0.05; *P*<.01), supporting H1. We used PROCESS macro (Model 7) to test the hypothesized first-stage moderated mediation model [75]. Specifically, we verified indirect effects using 5000 samples with bias-corrected bootstrap estimates to generate 95% CIs. To ensure our sample size provided sufficient statistical power to detect the hypothesized mediation effects, a power analysis was performed using the MedPower tool (David A. Kenny) [76]. Based on a sample of 450 and the observed path coefficients, the results indicated that the statistical power for detecting the indirect effects in both mediation models was virtually 1.00, well above the recommended 0.80 threshold. H2 and H3 proposed that bridging social capital and bonding social capital mediate the relationship between online social interaction and the intention to donate. As Table 5 shows, online social interaction was positively related to bridging social capital (*β*=0.45, SE 0.05; *P*<.01) and bonding social capital (*β*=0.54, SE 0.05; *P*<.01). Moreover, bridging social capital (*β*=0.48, SE 0.05; *P*<.01) and bonding social capital (*β*=0.54, SE 0.05; *P*<.01) were positively related to intention to donate. Furthermore, the PROCESS results indicated that bridging social capital partially mediated the relationship between online social interaction and intention to donate, as the direct effect remained significant (*β*=0.18, SE 0.05; *P*<.01). In contrast, bonding social capital fully mediated this relationship, as the direct effect of online social interaction on intention to donate became non-significant (*β*=0.11, SE 0.06; *P*=.06). Therefore, both H2 and H3 were supported.

**Table 5.** PROCESS results for the overall model (N=450).

Variables	Bridging social capital		Bonding social capital		Intention to donate	
	M1	M2	M3	M4	M5	M6
	Estimate (SE)	Estimate (SE)	Estimate (SE)	Estimate (SE)	Estimate (SE)	Estimate (SE)
Constant	—^a^	3.47^b^ (0.42)	—	3.13^b^ (0.36)	1.33^c^ (0.45)	1.46^d^ (0.45)
Online social interaction	0.45^b^ (0.05)	0.44^b^ (0.05)	0.54^b^ (0.05)	0.48^b^ (0.05)	0.18^b^ (0.05)	0.11 (0.06)
Bridging social capital	—	—	—	—	0.48^b^ (0.05)	—
Bonding social capital	—	—	—	—	—	0.54^b^ (0.05)
Moderation variables	—	—	—	—	—	—
Neighborhood perception	—	0.17^b^ (0.05)	—	0.31^b^ (0.05)	—	—
Interaction Int 1^e^	—	0.17^c^ (0.05)	—	0.18^c^ (0.05)	—	—
Control variables	—	—	—	—	—	—
Sex	0.06 (0.09)	0.17 (0.08)	−0.07 (0.08)	−0.06 (0.07)	0.13 (0.08)	0.25 (0.08)
Age	0.01 (0.06)	−0.02 (0.06)	0.04 (0.06)	−0.01 (0.05)	0.05 (0.06)	0.03 (0.06)
Income	−0.02 (0.04)	−0.01 (0.04)	−0.08 (0.04)	−0.07 (0.03)	−0.01 (0.04)	0.02 (0.04)
Education	−0.05 (0.06)	−0.06 (0.06)	−0.09 (0.05)	−0.11 (0.05)	−0.08 (0.05)	−0.05 (0.05)
Census register	−0.06 (0.09)	−0.07 (0.08)	−0.01 (0.08)	0.03 (0.07)	0.08 (0.08)	0.04 (0.08)
Political identity	−0.08 (0.12)	−0.15 (0.11)	−0.01 (0.11)	−0.02 (0.10)	0.01 (0.11)	−0.05 (0.11)
Marital status	−0.13 (0.13)	−0.13 (0.12)	−0.05 (0.11)	−0.04 (0.11)	−0.06 (0.12)	−0.10 (0.12)
*R* ^2^	0.23	0.26	0.32	0.41	0.32	0.33
*F* test (*df*)	16.04 (8, 441)^b^	15.59 (10, 439^b^	25.94 (8, 441)^b^	30.03 (10, 439)^b^	23.45 (9, 440)^b^	23.97 (9, 440)^b^

a Not applicable.

b Statistically significant at the .001 level.

c Statistically significant at the .01 level.

d Statistically significant at the .05 level

e Int 1=online social interaction × neighborhood perception; unstandardized coefficients of PROCESS.

**Figure 2.**
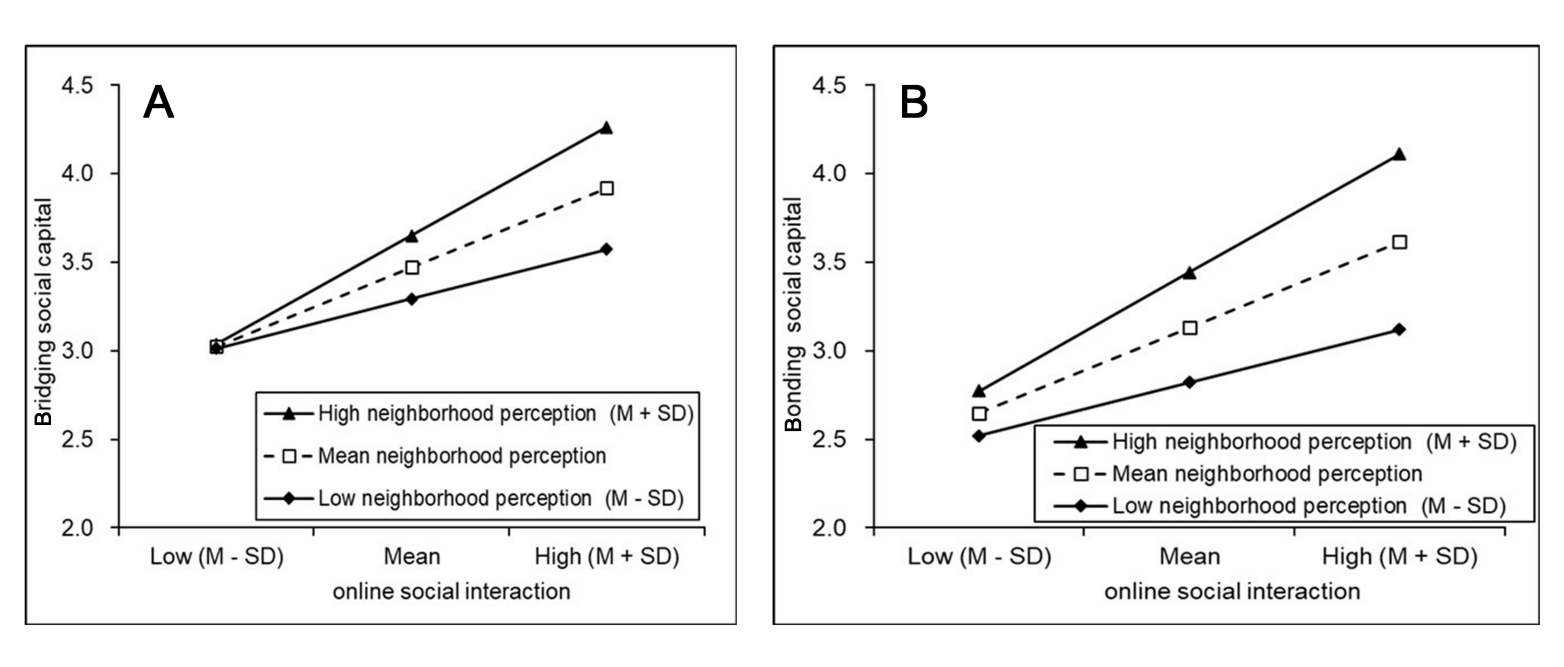
Moderating effect of neighborhood perception on the relationship between online social interaction and bridging social capital (A) and bonding social capital (B).

The PROCESS results also provided empirical support for H4 and H5, which proposed that individuals’ neighborhood perceptions moderate the relationship between online social interaction, bridging social capital, and bonding social capital. Given that H4 and H5 included interaction, prior to the construction of the interaction term (neighborhood perception × online social interaction), online social interaction and neighborhood perception were mean-centered. As shown in Table 5, the results indicated that the interaction term was positively associated with bridging social capital (*β*=0.17, SE 0.05; *P*<0.01) and bonding social capital (*β*=0.18, SE 0.05; *P*<.01). Additionally, a simple slope test revealed that when the level of neighborhood perception was low, online social interaction was positively associated with bridging social capital (*β*=0.31, SE 0.07; *P*<.01), and this relationship became stronger when neighborhood perception was high (*β*=0.58, SE 0.06; *P*<.01). Similarly, when neighborhood perception was low, online social interaction was positively associated with bonding social capital (*β*=0.34, SE 0.07; *P*<0.01), and the relationship was stronger at higher levels of neighborhood perception (*β*=0.63, SE 0.05; *P*<.01). Thus, H4 and H5 were supported [77] (Figure 2A and B).

H6 and H7 proposed that individuals’ neighborhood perceptions enhance the indirect effects of online social interaction on their intention to donate via bridging and bonding social capital. As shown in Table 6, the conditional indirect effects were significant when neighborhood perception was high (*β*=0.278, 95% CI 0.195-0.362 for bridging social capital; *β*=0.340, 95% CI 0.257-0.424 for bonding social capital). In contrast, although still significant, these effects were weaker when neighborhood perception was low (*β*=0.150, 95% CI 0.059-0.248 for bridging social capital and *β*=0.181, 95% CI 0.097-0.270 for bonding social capital). Thus, H6 and H7 were supported. Moreover, the moderated mediation index provided further evidence for these moderated mediation effects [78], showing significance for intention to donate via bridging social capital (*β*=0.080, 95% CI 0.021-0.142) and bonding social capital (*β*=0.100, 95% CI 0.040-0.162). Notably, the moderated mediation effect was stronger for the bonding social capital pathway than for the bridging pathway.

**Table 6.** Conditional indirect effects of online social interaction on intention to donate via online social capital moderated by neighborhood perception (N=450)^a^.

Mediation and levels of EP^b^ (moderator)	Indirect effect (95% CI)	Index of moderated mediation (95% CI)
Bridging social capital		0.080 (0.021-0.142)
Mean +1 SD	0.278 (0.195-0.362)	
Mean	0.214 (0.142-0.291)	
Mean −1 SD	0.150 (0.059-0.248)	
Bonding social capital		0.100 (0.040-0.162)
Mean +1 SD	0.340 (0.257-0.424)	
Mean	0.260 (0.193-0.331)	
Mean −1 SD	0.181 (0.097-0.270)	

a Statistical significance is indicated when the CI does not include zero.

b EP: neighborhood perception.

**Figure 3.**
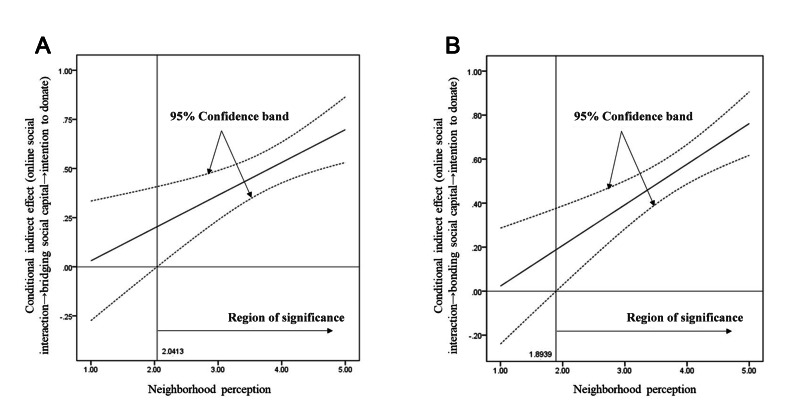
Conditional indirect effect of online social interaction on intention to donate via bridging social capital (A) and bonding social capital (B) at values of neighborhood perception.

Furthermore, the Johnson-Neyman technique [79] was used to identify regions of significance for the conditional indirect effects. As depicted in Figure 3A and B, these effects were not significant at low levels of neighborhood perception. Given the 5-point Likert scale, the Johnson-Neyman thresholds allow substantive interpretation. For bridging social capital, the indirect effect was significant for neighborhood perception values above 2.04, indicating that its mediating role emerges only from low to moderate levels onward. For bonding social capital, the threshold was lower (1.89), suggesting that its mediating effect is activated even at slightly below-moderate levels. This difference suggests that bonding social capital has a lower activation threshold due to its reliance on close, trust-based ties, whereas bridging social capital requires a more supportive perceived environment. Accordingly, when neighborhood perception is low, online social interaction is less likely to translate into donation intention, while at moderate to higher levels, both forms of social capital become effective pathways.

### Analysis of Control Variables and Moderation Effects

To enhance the robustness of our findings, we examined the direct effects of control variables (age, income, education, gender, and residency) on the primary constructs. As presented in Table 7, only gender significantly predicted intention to donate (*β*=0.120, SE 0.038, *P*<.01), whereas other demographic factors remained nonsignificant. Furthermore, we conducted a multigroup SEM analysis to explore the potential moderating effect of gender, the only significant control variable. We compared a configural model (unconstrained) with a structural model where all path coefficients were constrained to be equal across groups. The results of the chi-square difference test (Table 8) indicated that the model fit did not significantly decrease after applying these constraints (*Δχ*²_4_=8.523, *P=.*07). This suggests that the relationship between the independent and dependent variables is stable and does not vary significantly by gender, confirming the generalizability of our hypothesized model.

**Table 7.** Direct effects of control variables on ITD^a^ (*P*<.01).

Control variables	β (SE)	*z* value	*P* value
Age	0.023 (0.041)	0.547	.58
Monthly income	−0.003 (0.041)	−0.072	.94
Education	−0.038 (0.038)	−0.995	.32
Gender	0.120 (0.038)	3.187	.001
Residence	0.037 (0.038)	0.983	.33

a ITD: intention to donate.

**Table 8.** Multigroup SEM invariance test for gender.

Model	Chi-square (*df*)	Δ Chi-square ^a^(Δ*df*)	*P* value
Unconstrained model	1620.233 (770)	—^b^	—
Constrained model	1628.756 (774)	8.523 (4)	.07

a ΔChi-square is the difference in chi-square values between the constrained and unconstrained models.

b Not applicable.

## Discussion

### Principal Findings

The results indicate that online social interaction is significantly associated with donation intentions, operating through both online bridging and bonding social capital. Rather than implying a causal mechanism, these findings suggest that the intention to donate to patients with serious illnesses may involve a dual-path associative process in which both weak and strong ties play a role. This result is consistent with prior studies [12,23], which have highlighted the positive roles of bridging and bonding social capital in shaping prosocial outcomes.

Further, the finding that bonding social capital fully mediates the relationship between online social interaction and donation intention, whereas bridging social capital shows partial mediation, suggests that emotional closeness plays a dominant role in health-related charitable decision-making. In the context of serious illness, individuals are frequently exposed to emotionally salient narratives that emphasize vulnerability, suffering, and hope. Such illness narratives tend to elicit empathy and emotional resonance, which strengthen affective bonds and ultimately motivate prosocial behaviors such as donation [80]. This mechanism can be understood through the relational nature of bonding social capital, which is characterized by emotional intensity, trust, and strong interpersonal ties [17,18]. These affective qualities are particularly salient in health charity contexts, where moral obligation and empathic concern are key drivers of helping behavior. In contrast, bridging social capital primarily facilitates access to heterogeneous information and weak-tie connections, which may increase awareness but lack the emotional depth required to fully translate into donation intention [18,35]. This distinction explains why bonding social capital exerts a full mediating effect, whereas bridging social capital plays a complementary but partial role. Furthermore, this finding aligns with emerging eHealth literature emphasizing the role of affective engagement in digital health environments. Studies on digital peer support and patient-centered applications suggest that emotionally responsive systems can enhance empathy-driven interaction and prosocial engagement [77,79,81]. These findings suggest that digital prosocial behavior in health care volunteer communities is not primarily driven by information exposure alone, but by emotionally embedded and trust-centered relational processes [12,24,82]. In this context, online interaction becomes a form of relational support through which empathy and emotional connection are cultivated, thereby motivating charitable engagement [81,83]. This finding extends existing social capital research by highlighting the emotional and relational embeddedness of digital altruism in health care–related settings. Additionally, the stronger effect of bonding social capital compared with bridging social capital suggests that digitally mediated charitable intentions in health care settings are sustained more by emotionally dense and trust-based relational ties than by weak informational connections [23,24]. This finding further indicates that perceived intimacy and affective solidarity function as important motivational foundations for prosocial engagement in digitally mediated health care communities [81,83]. Overall, these findings suggest that online health care volunteer communities operate not merely as informational networks, but as affective and relational infrastructures that sustain digital prosocial engagement.

This study also demonstrates that neighborhood perception moderates the indirect effect of online social interaction on donation intention through both bridging and bonding social capital. These findings provide a complementary perspective for understanding how neighborhood perception and online interaction jointly shape prosocial engagement in the context of serious illness care charity. Specifically, the 2 pathways appear to reflect distinct yet complementary social mechanisms. The pathway through bridging social capital is primarily associated with norm-based processes, whereby individuals with stronger neighborhood perception become more sensitive to social expectations, reputational concerns, and norms of prosocial behavior [16]. This heightened norm awareness encourages engagement in broader, weak-tie networks and facilitates access to broader social support and informational resources, thereby strengthening donation intention. In contrast, the pathway through bonding social capital is more closely linked to relationship-based processes, in which stronger neighborhood perception enhances interpersonal trust, emotional connection, and a sense of belonging [54]. These relational dynamics foster emotionally supportive and reciprocal ties among donors, volunteers, and patients, thereby reinforcing prosocial motivation and donation intention. Overall, these findings highlight that offline neighborhood perception and online social interaction are not independent influences, but mutually reinforcing relational environments that shape digital prosocial engagement.

### Theoretical Contributions

This study contributes to the literature by deepening our understanding of how online interactions and offline contexts jointly shape digital altruistic behavior within health care volunteer communities. This study further expands social capital research, revealing the distinct roles that bridging social capital and bonding social capital play in shaping the willingness to donate [23,24]. Although both forms of social capital are positively associated with donation intention, bonding social capital exerts a stronger influence. This suggests that digitally mediated charitable engagement is based more on emotionally intense, trust-based relationships than on weak informational ties [12,81]. In this sense, this study offers a new perspective on understanding the relational and emotional embeddedness of digital altruistic behavior in health care–related contexts [14,15].

This study also expands the body of research on online interaction, moving beyond the informational perspective of digital communication [11,12]. And this study does not view online interaction merely as the exchange of information, but rather regards active and constructive online participation as a form of relational support that fosters trust, empathy, and emotional connection through this process [15]. This perspective complements existing research on online social capital by emphasizing the relational processes through which digital prosocial engagement becomes socially embedded [14,81].

In addition, this study advances the literature by incorporating neighborhood perception as an important offline contextual condition shaping the formation of online social capital [23,24]. While prior studies have primarily focused on platform characteristics or online behavioral factors, this study highlights the socio-spatial embeddedness of digital social capital formation. More specifically, the findings suggest that neighborhood perception simultaneously reinforces norm-based processes associated with bridging social capital [16] and relationship-based processes associated with bonding social capital [54]. These findings indicate that digital prosocial engagement is not formed solely within online environments but emerges through the interaction between online relational dynamics and offline social contexts. Overall, this study provides an integrated theoretical perspective on how online interaction, neighborhood perception, and differentiated forms of social capital jointly shape prosocial engagement in digitally mediated health care communities.

### Implications for eHealth Tool Development

The identified mechanisms can be translated into actionable strategies for digital platforms. Our findings highlight the critical role of platform affordances in facilitating social capital accumulation. The structure of digital platforms, such as algorithmic content feeds, notification systems, and interactive communication channels, can foster a sense of community and accountability [84]. For instance, algorithm-driven exposure and timely reminders may reinforce weak ties and enhance bridging social capital, while interactive functions such as private messaging and group discussions can strengthen trust and emotional attachment, thereby fostering bonding social capital.

The dual-path mechanism suggests that eHealth platforms should adopt differentiated engagement strategies. Bridging social capital can be promoted through broad information dissemination, open networks, and low-threshold participation. In contrast, bonding social capital may be strengthened through personalized storytelling (eg, patient narratives and volunteer experiences), which can foster empathy and sustained engagement. Prior research has shown that character-driven narratives in digital environments increase narrative transportation and subsequent donation behavior [80].

These findings support the integration of digital “nudges” into eHealth systems. Prior research has shown that peer support, social interaction, and behavior change techniques in mHealth interventions can enhance user engagement and promote sustained participation [84-86]. Accordingly, subtle design interventions, such as personalized recommendations, emotionally engaging prompts, and context-sensitive notifications, can encourage prosocial behaviors with minimal cognitive burden. These approaches are particularly valuable in mobile health environments, where sustaining engagement remains a key challenge.

Furthermore, this study reframes the “3C” Health Care Volunteer Community as a prototype of a digital health intervention, which integrates online interaction, social capital formation, and prosocial health engagement. This perspective highlights the potential of digitally mediated communities as scalable models for fostering peer support and charitable participation in health contexts. As online social interaction and charitable engagement increasingly rely on platform-mediated communication, issues such as data security, user consent, and the responsible use of personal health information become critical, particularly in health-related crowdfunding and online volunteering platforms. Ensuring transparency and trust in data governance is essential for sustaining user engagement and fostering long-term participation in digital health communities.

### Practical Implications

Extending these findings to digital health contexts, platform-based interventions can be strategically designed to enhance user engagement and prosocial behavior. To encourage donation intention, community operators can provide multiple channels to stimulate active social interaction, for example, interactions between public accounts and online communication groups. Beyond purely online strategies, greater emphasis should be placed on integrating online platforms with offline community infrastructures. For example, linking neighborhood-based applications with widely used social platforms such as WeChat can facilitate real-time coordination, lower participation barriers, and enable localized health volunteering within familiar social networks.

Offline-online synergies can be further strengthened by embedding digital participation into everyday community health touchpoints, linking offline care events with sustained online interaction, and developing data-driven feedback loops that inform local health governance. In addition, integrating spatially grounded community resources (eg, health centers, therapeutic gardens, and neighborhood activity spaces) with digital platforms can facilitate context-aware volunteering and enhance both bonding and bridging social capital. Given the mediating effect of social capital, importance should be attached to building strong and weak ties across online and offline settings. Active interaction among participants in charitable donations and sustained online contact between volunteers and patients following offline care activities can strengthen emotional connections and reinforce trust. Broader online connections across diverse social groups can facilitate information exchange, diffusion of prosocial norms, and resource mobilization. The interplay between offline encounters and online communication thus creates a reinforcing loop that enhances both bonding and bridging social capital, ultimately promoting sustained engagement in digital health volunteering.

The moderating role of neighborhood perception further highlights the importance of offline community environments in shaping digital prosocial behavior. Safer and more cohesive neighborhoods can foster trust and social interaction, which in turn strengthens both online bridging and bonding social capital. This suggests that improving neighborhood conditions, such as enhancing safety, promoting social cohesion, and encouraging local participation, can indirectly support digital health engagement. Moreover, the roles of schools, businesses, and other venues should also receive increased focus.

### Limitations and Future Research Directions

First, this study is based on cross-sectional data, which limits causal inference. Sensitivity analyses (eg, instrumental variables or longitudinal simulations) could strengthen causal interpretation but require different data structures and were beyond the scope of this study. Therefore, the observed mediation patterns should be interpreted with caution. Future research should use longitudinal or experimental designs and appropriate sensitivity analyses to better address causality and potential endogeneity.

Second, reliance on self-reported measures of donation intention may introduce CMB and social desirability bias. In the context of charitable giving, respondents may overstate their willingness to donate due to normative expectations or perceived social approval. This may lead to an overestimation of the relationships observed in this study. Future research could complement self-reported intentions with behavioral data (eg, actual donation records or platform trace data) or adopt more indirect measurement techniques to reduce such bias. In addition, the dependent variable in this study is donation intention rather than actual donation behavior. Although intention is a theoretically meaningful predictor of behavior, it may not always translate into action. Therefore, the findings should be interpreted with caution when extending to real-world donation behavior.

Third, the sample is relatively homogeneous, mainly comprising young female participants from Guizhou, China, which may limit generalizability to more diverse eHealth populations across different age groups and sociocultural settings. In addition, participants were recruited through health care volunteer-related networks and represented varying levels of engagement with the 3C community, ranging from active volunteers to peripheral supporters and individuals familiar with related charitable initiatives. Although this diversity of engagement provides a meaningful context for examining digitally mediated prosocial behavior, the findings should be interpreted with caution when generalizing to broader populations. Future studies should adopt multisite designs and more diverse samples to enhance external validity.

Future research could use experimental designs to strengthen causal evidence. In particular, A/B testing of online interaction environments (eg, varying emotional framing, peer visibility, social endorsement cues, or interaction intensity on crowdfunding platforms) may help disentangle how different forms of online social interaction influence the formation of social capital and subsequent donation intentions.

### Conclusion

Online social interaction was associated with donation intentions toward patients with serious illness via bridging and bonding social capital, with bonding ties fully mediating the effect and bridging ties partially mediating it. Neighborhood perception strengthened these pathways, suggesting that offline neighborhood context can enhance the impact of online engagement on charitable giving. These findings highlight the importance of integrating digital interaction with offline environments in promoting health-related charitable behavior and provide practical implications for designing digital health tools.

## Abbreviations

3C: Caring for Children with Cancer
AVE: average variance extracted
CLF: common latent factor
CMB: common method bias
